# Long-Term Treatment Outcomes of Implant Prostheses in Partially and Totally Edentulous Patients

**DOI:** 10.3390/ma15144910

**Published:** 2022-07-14

**Authors:** Eugenio Velasco-Ortega, Inmaculada del Rocío Jiménez-Martin, Jesús Moreno-Muñoz, Enrique Núñez-Márquez, José Luis Rondón-Romero, Daniel Cabanillas-Balsera, Álvaro Jiménez-Guerra, Iván Ortiz-García, José López-López, Loreto Monsalve-Guil

**Affiliations:** 1 Department of Comprehensive Dentistry for Adults and Gerodontology, Faculty of Dentistry, University of Seville, 41009 Seville, Spain; evelasco@us.es (E.V.-O.); inma_rocio@hotmail.com (I.d.R.J.-M.); je5us@hotmail.com (J.M.-M.); enrique_aracena@hotmail.com (E.N.-M.); jolurr001@hotmail.com (J.L.R.-R.); danielcaba@gmail.com (D.C.-B.); ivanortizgarcia1000@hotmail.com (I.O.-G.); lomonsalve@hotmail.es (L.M.-G.); 2 Department of Odontostomatology, Medicine and Health Sciences, Dentistry, Master of Oral Medicine, Oral Surgery and Oral Implantology, Service of the Medical-Surgical Area of Dentistry Hospital, University of Barcelona, 08907 Barcelona, Spain

**Keywords:** dental implants, long-term treatment, implant prostheses, implant complications, peri-implantitis, implant failure, prosthetic complications, implant complications, type of implant connection

## Abstract

Implant dental therapy is a clinical procedure used for treating patients with tooth loss with known clinical success. This clinical study aimed to evaluate the long-term clinical outcomes of dental implants in partially and totally edentulous patients. A total of 544 Microdent (Microdent SU, Implant Microdent System^®^, Santa Eulàlia de Ronçana Barcelona, Spain) screw implants were placed in 111 patients using a two-stage surgical technique and a conventional loading protocol (lasting 3 months). Implant and prosthetic clinical findings were evaluated during a 15-year follow-up. A total of 6 implants were lost during the healing period, and 124 prostheses were placed over the 538 implants that remained: 20 single crowns, 52 partially fixed bridges, 45 full-arch fixed restorations, and 7 overdentures. A total of 20 of these were lost during the follow-up period. The cumulative survival rate for all implants was 96.4%. The data underwent statistical analysis (significance level: *p* < 0.05). The mean marginal bone loss was 1.82 ± 0.54 mm, ranging from 1.2 to 3.1 mm. The most frequent complications were mechanical prosthodontic complications (16.2%). In all, 11.8% of implants showed periimplantitis as the primary biological complication. Dental implants inserted in both the maxillary and mandibular areas produce long-term favorable outcomes and stable tissue conditions when a delayed loading protocol is followed.

## 1. Introduction

Implant therapy is a widely used treatment in patients suffering from dental loss. Implant-supported prostheses are often used in the rehabilitation of partially or totally edentulous patients [1,2]. The clinical success of dental implant therapy is directly related to a successful osseointegration process, which is the consequence of a series of biological processes that occur after the surgical insertion of a dental implant into the alveolar bone [3]. The industry and clinics actively research innovative materials and technologies to improve treatment outcomes, simultaneously reducing morbidity, as well as biological and surgical times [4].

The influence of implant-surface and macroscopic design characteristics on the long-term results of dental implants is very important and has been discussed recently. Today, with the improvement of surface treatments, the osseointegration process is highly successful, and it is directly related to the biologic connection between the implant surface and the host bone tissue [3,5]. The dental implant’s surface features, such as its roughness and chemical composition, can cause different biological responses and may increase the contact area between the implant and the surrounding bone, that is, the bone-to-implant contact (BIC), improving the biocompatibility and enhancing the osseointegration [6,7,8,9].

Macroscopic design, especially that of the implant–abutment connections, has been considered as important for the success of dental implant treatment. External joints are a common feature in implant–abutment connections [10]. During mastication, the dental implant interacts with compressive force, which is parallel to the implant’s long axis and shearing force, which is not perpendicular to the implant’s long axis. In fact, an important survival criterion for dental implant treatment is the behavior of the implant–abutment connections in the oral cavity, especially for mastication, despite the existence of bone loss during the functional period [11].

Restorative reconstructions performed on implants show high success and survival rates. Prosthetic-supported-implants are a treatment option commonly used for restoring partially and totally edentulous arches [12,13]. When the edentulous areas are accompanied by moderate or severe bone loss, several types of prosthodontic restorations can be considered as treatment options for restoring the dentition with favorable, functional, and aesthetic success [14]. Many studies have documented that reconstruction by implant-supported single crowns or fixed partial bridges represents a persistent, preventive tool to rehabilitate partially edentulous patients. In totally edentulous patients, implant-supported, fixed prostheses or removable overdentures are available and present reliable solutions for rehabilitation [15,16].

The number of long-term clinical studies assessing the outcomes of implant treatment for more than 15 years is limited, and clinicians and patients may benefit from knowledge about the findings regarding implants and prostheses in the long-term [17,18,19]. To further optimize the long-term success rates of dental implants, a better understanding of the frequency and nature of implant failures, together with their potential contributing factors, is essential. Despite relatively high success rates, implant treatment failures do occur over time, either due to peri-implant infections, progressive bone loss, or loss of osseointegration [17]. Also, the criteria for success at the prosthetic level, along with the occurrence of technical complications/prosthetic maintenance and adequate function, must be incorporated. Success in implant dentistry should ideally evaluate the long-term outcomes of an implant–prosthetic complex as a whole [20,21].

The objective of this clinical study was to evaluate the long-term clinical outcomes of implant-supported-prostheses, including implant survival with marginal bone conditions over time, as well risk factors for biological and technical complications. Patient factors were evaluated for the risk of failure, including periodontal disease, smoking, and medical conditions.

## 2. Material and Methods

### 2.1. Sample Description

This study included patients with partial or total edentulism who required treatment with dental implants. All surgeries and prosthetic procedures were developed in the School of Dentistry of the University of Seville, Spain, from November 2005 to January 2012. The study was conducted according to the principles outlined in the Declaration of Helsinki [22] for clinical research involving humans. All patients signed a double-informed, written consent for implant placement and to be part of the clinical study. The ethical committee of the University of Seville approved the study [Ethics Committee validated in Seville, Spain, on 3 March 2022, Responsible Prof. José María Llamas Carreras].

#### Inclusion and Exclusion Criteria

The inclusion criteria included having reached adulthood, having a good systemic health status (ASA I or II) or controlled systemic diseases, and the lack of a need for bone-regeneration techniques.

The exclusion criteria were the presence of uncontrolled, chronic, systemic disease (diabetes or cardiovascular disease), smoking more than ten cigarettes per day, coagulation disorders, alcohol or drug abuse, and the use of any medication or health alteration that contraindicated implant treatment.

### 2.2. Diagnosis and Treatment Plan

**Figure 1:**
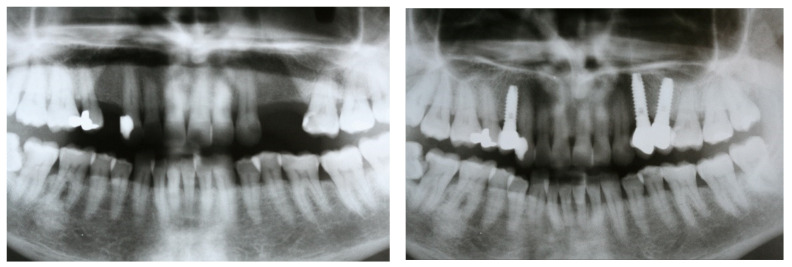
Panoramic radiographs were taken before treatment as part of the diagnosis and treatment plan and after implant placement and prostheses delivery.

Treatment planning included making diagnostic casts to evaluate intermaxillary relations, clinical photographs, and panoramic radiographs (Figure 1). Most of the patients (especially since 2010) were evaluated with computerized tomography when required.

### 2.3. Surgery Protocol

“All patients received prophylactic antibiotic therapy 1 h before surgery (amoxicillin 500 mg and clavulanic acid 125 mg) which continued to be administered every 8 h for 7 days. In the postoperative period, 600 mg of ibuprofen was also prescribed: every 8 h for 3 days, and then according to demand for pain”. The use of chlorhexidine (Bexident^®^ Post, Barcelona, Spain) mouthwash was recommended twice-daily for 1 month. All patients were treated under local anesthesia with articaine and adrenaline (40 mg/mL + 5 micrograms/mL).

A mucosal-flap approach was made, and the implants were inserted in the selected places, following a prosthodontic guided plan. The drilling protocol was that recommended by the manufacturer (Implant Microdent System^®^, Santa Eulàlia de Ronçana, Barcelona, Spain), and the minimum insertion torque was 35 Ncm. All implants were inserted with a delay, in healed bone with a two-stage surgical technique. No bone or soft tissue grafts were applied.

**Figure 2:**
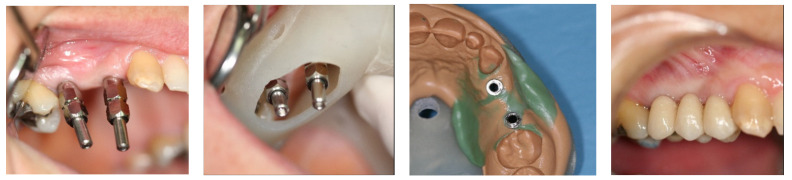
Prosthetic clinical protocol: Impression coping at 3 months post-placement and final, fixed-bridge delivery.

Secondary-stage surgery was performed 3 months after implant placement, and healing or prosthetic abutments were placed. Functional loading was completed when the insertion torque achieved at least 35 Ncm. Definitive prostheses were placed 3 weeks (±3 days after the second stage of the surgery (Figure 2).

### 2.4. Follow-up

Follow-up visits were scheduled at 3- and 6-months after prosthesis placement and every year during a mean period of 14.54 ± 1.1 years (ranging between 10 and 16.1 years). The success criteria were established as implant stability and the absence of radiolucency around the implant, mucosal suppuration, or pain. Marginal bone loss was determined by an intraoral, digital radiograph taken perpendicular to the long axis of the implant.

### 2.5. Implant Characteristics

Microdent screw implants (Microdent SU, Implant Microdent System^®^, Microdent Universal, External Hex Connection, Santa Eulàlia de Ronçana, Barcelona, Spain) were used for all patients. The implant surface was treated with sandblasting, which is a surface treatment designed to increase the surface roughness, inducing sub-micro topography without leaving residual, embedded blast particles or debris on the treated surface.

The implant was a tissue-level, commercially pure (CP) titanium, grade IV implant, characterized for an external hexagon connection, with a wide head-shape, without microthreads, and with V-shaped threads in the body, a straight body-shape, and a dome-apex shape.

### 2.6. Statistical Evaluation

SPSS 18.0 software (SPSS Inc., Chicago, IL, USA) was used for data evaluation. Descriptive statistics of the clinical findings of the study were performed with reference to the demographic and clinical variables of the patients, the characteristics of the implants (success, complications, and losses), the conventional functional load, and the prosthodontic restorations performed. Contingency tables were made of all the qualitative variables that were analyzed with a chi-squared test in each cell: frequency and percentage according to columns.

The quantitative variables were analyzed according to the variance test when the distribution was normal, with respect to all of the qualitative variables. We performed a non-parametric test of non-normally distributed, numerical variables with respect to all of the qualitative variables. A Mann–Whitney U test was used for dichotomous variables, and the Kruskal–Wallis test was used for variables with more than two categories.

Statistical significance was established with a *p* < 0.05.

## 3. Results

A total of 544 implants were placed in 111 partially and totally edentulous patients: 56 males and 55 females. The demographic distribution of the 111 patients included in the study was: 56 males and 55 females with ages ranging from 32 to 74 years old, with a mean age of 53.1 (SD = 9.6) years old. No significant statistical differences were found to be related to sex or age (chi-square test, *p* = 0.06420). A total of 35 patients (31.5%) were totally edentulous, and 76 (68.5%) patients were partially edentulous.

A total of 46 patients (41.4%) had a previous history of periodontitis, 56 patients (50.4%) were smokers, and 67.3% of the patients with a previous history of periodontitis were smokers (*n* = 31) (Table 1).

Of the 544 implants placed, 90 (16.5%) had a diameter of 3.5 mm, 408 (75%) had a diameter of 4 mm, and 46 (8.5%) had a diameter of 5 mm. The most significant data are presented in Table 2.

Regarding the prostheses designed, a total of 124 prostheses were placed in the 111 patients over the 538 remaining implants after the healing period (3 months). The prostheses were distributed in the following way: In essence, 20 single crowns were placed in 15 patients; 52 partially fixed-bridges supporting from 2 to 4 implants were placed in 46 patients over a total of 144 implants; 45 full-arch fixed restorations were placed in 43 patients (2 patients were full-bimaxillary edentulous) over a total of 346 implants; and 7 overdentures were placed in 7 full-mandibular edentulous patients over a total of 28 implants (see Table 3).

The mean marginal bone loss was 1.82 mm (SD = 0.54 mm), ranging from 1.2 to 3.1 mm during the time interval from the implant insertion to the 15-year follow-up evaluation. In patients with a previous history of periodontitis, this marginal bone loss was 1.92 ± 0.50, while in patients without periodontitis, it was 1.75 ± 0.55. These differences show statistical significance (ANOVA; *p* = 0.0088). Regarding the smoking habit, the marginal bone loss was 1.92 ± 0.55 for smoking patients and 1.72 ± 0.50 for non-smoking patients, with statistical differences (ANOVA; *p* = 0.0017). A smoker was considered as such when the clinical history so specified, taking into account that the criterion used in the work group that carried out the study was to consider a patient as a smoker when they smoked daily or consumed more than 10 cigarettes per week.

During the follow-up period, 78 implants (14.3%) of the 538 remaining implants were associated with peri-implantitis, with 14 of them being lost. These implants were classified as delayed failures. Peri-implantitis was more frequent, showing statistically significant differences in those patients with a previous history of periodontitis (41.4%); only 18% of the patients without previous periodontitis developed peri-implantitis (chi-square test, *p* = 0.01350). Peri-implantitis was also more frequent in smokers (42.9%); only 18.2% of non-smokers developed peri-implantitis, with statistically significant differences (chi-square test, *p* = 0.00481). A total of 18 patients (16.2%) showed some kind of prosthodontic complications (loss/fracture of the prosthetic screw, ceramic chipping, or resin fracture) in 76 of the 538 remaining implants. A total of 1 crown, 6 fixed-partial bridges, 10 fully fixed rehabilitations, and 1 overdenture had to be renewed (Table 4).

## 4. Discussion

The present, retrospective study reports on the survival rate of dental implants with an external connection and a sandblasted surface in partially and totally edentulous patients. Our results yielded an implant survival rate of 96.3% in delayed loaded implants. A total of 111 patients received dental implants in the School of Dentistry, University of Seville, and had a 15-year follow-up. Only patients with a good quality and quantity of bone were selected to be part of this study, so no grafting materials or barrier membranes were used. The clinical protocol included a submerged surgical technique, and loading was performed after 3 months of healing.

Implants with external connections were used in several long-term follow-up studies on the treatment of implant-supported, fixed prostheses in edentulous patients. The results, after more than 20 years, demonstrated an important cumulative survival/success rate for the implants [23,24]. A clinical study showed successful results with the same type of implants inserted with a conventional technique or with a minimally invasive technique, without a mucoperiosteal flap elevation (i.e., flapless) [25]. A total of 48 implants were placed in 30 patients (15 participants per group). In this study, only one implant placed in a conventional surgery group (2.1%) failed before prosthetic loading due to mobility and pain at the 3-months follow-up. The implant survival rate was 97.9% [25].

The implant’s surface topography plays an essential role in the osseointegration process. Nowadays, most implants are moderately rough, and their surfaces have been treated by sandblasting, acid-etching, anodization, or other techniques. Sandblasted surfaces improve the adherence, migration, and proliferation of osteoblastic cells [9,26]. This long-term, clinical study shows excellent outcomes for treatments performed with sandblasted implant surfaces. Similar results were reported in several studies that evaluated the clinical success of sandblasted implants [27,28,29].

In the present study, 94.4% of the prostheses were fixed (single-crown, fixed-bridge, or full-arch rehabilitation), and 5.6% were removable prostheses (overdentures). The clinical findings in this long-term follow-up study suggest that implants inserted in both maxillary and mandibular areas produce favorable outcomes and stable tissue conditions when a delayed loading protocol is followed. Rasmusson et al. [30] in a prospective study confirmed these results after the evaluation of submerged, sandblasted implants after 10 years of prosthetic loading. A total of 199 implants were placed in 36 patients. Of those, 91 were placed in the upper jaw, and 108 implants were inserted in the lower jaw. Fixed prostheses were delivered after a healing period of 3 to 6 months. A total of 6 implants failed during the first year of follow-up (3 in the mandible and 3 in the maxilla), giving a cumulative survival rate of 96.9% [30].

Our results show a marginal bone loss of 1.82 ± 0.54 mm, ranging from 1.2 mm to 3.1 mm after a 15-year follow-up. This bone loss was higher than the results described in other long-term clinical studies [15,26,30]. Östman et al. [30] found marginal bone loss of 0.7 ± 1.35 mm. However, Astrand et al. [23] reported the outcome of implant treatment with fixed prostheses in edentulous jaws after 20 years, showing a mean bone loss of 2.33 mm at the final examination. The high level of bone loss in the present study might be explained by the fact that many patients included in this study were smokers (50.4%) and showed a previous history of periodontal disease (41.4%).

Prosthetic complications occurred frequent in this study. a total of 18 patients (16.2%) showed some kind of technical complication: ceramic chipping, loss or fracture of the prosthetic screw, or acrylic fracture. Of 124 prostheses, 10 fully fixed rehabilitations, 6 fixed bridges, 1 overdenture, and 1 single crown had to be restored or renewed, resulting in a prosthodontic survival rate of 85.5%. Technical complications are relatively common in other studies with long follow-up periods [1,16,18,29,30].

The most important biological complication in the present study was peri-implantitis. During the follow-up period, 78 implants (14.5%) were associated with this disease, and 14 implants were finally lost. Peri-implantitis was, significantly, more frequent in patients with a previous history of periodontitis (41.4%) and in smoking patients (42.9%). Smoking and a previous history of periodontal disease are identified as risk factors for peri-implantitis. The concomitant presence of smoking and a prior history of periodontitis increases the severity of peri-implantitis [31,32,33]. Previous studies on the long-term results of implant therapy reported an important prevalence of mucositis and peri-implantitis [34,35].

The present study has some limitations, such as the fact that it is a retrospective study and the implants were of different lengths and thicknesses, aspects that were considered at the same time to be advantages. However, our study also has some strengths, such as the broad sample involved in the study, the extended follow-up, the strict protocol followed during the surgeries and follow-up periods, the use of implants with external connections and the same types of surfaces (specifically sandblasted implant surfaces), and the variety of prosthesis types delivered in the prosthetic phase. Another limitation of the study is that there are no exact data on the level of the hygiene of the patients, but it should be considered that the clinical group performs annual maintenance on the patients.

## 5. Conclusions

Within the limits of the present long-term, follow-up, clinical study, we can conclude that the use of dental implants to support different types of prosthetic restorations results in successful treatment in regard to implant and prosthetic survival rates. The marginal bone loss was high, which was an expected finding, taking into consideration the high number of smokers and periodontal patients participating in this study. Moreover, biological and technical complications are frequent and constitute an important problem for both clinicians and patients.

## Figures and Tables

**Table 1:** Description of the sample distribution, according to the following parameters: type of edentulism, previous history of periodontitis, and smoking habit.

Patient Description *n* = 111 (100%)		
Type of Edentulism	Total	Partial
	*n* = 35 (35%)	*n* = 76 (68.5%)
Periodontitis History	Yes	No
	*n* = 46 (41.4%)	*n* = 65 (58.5%)
Smoking Habit	Smoker	Nonsmoker
	*n* = 56 (50.5%)	*n* = 55 (49.5%)
67.3% of patients with a previous history of periodontitis were also smokers (*n* = 31)		

**Table 2:** Distribution of the implant characteristics: diameter, length, location, area of placement, and preloading percentage of failure/success.

Implant Description		*n* = 544 (100%)	
Diameter	3.5 mm 90 (16.5%)	4 mm 408 (75%)	5 mm 46 (8.5%)
Length	10 mm 95 (17.5%)	11.5 mm 449 (82.5%)	
Location	Maxilla 405 (74.5%)	Mandible 139 (25.5%)	
Area	Anterior 238 (43.7%)	Posterior 306 (56.3%)	
Percentage of Failure/Success	Failure preloading 6 (1.1%)	Success preloading 538 (98.9%)	

**Table 3:** Description of the prosthesis-type distribution between the total number of patients and implants used to support them.

Prosthesis Type	Patients *n* = 111 (100%)	Implants *n* = 538 (100%)
Single crown	15 (13.5%)	20 (3.7%)
Fixed bridge	46 (41.5%)	144 (26.8%)
Full-arch fixed	43 (38.7%)	346 (64.3%)
Overdenture	7 (6.3%)	28 (5.2%)

**Table 4:** Description of the complications presented in the implants. * all losses were due to peri-implantitis; ^#^ Chi-square test; ^&^ ANOVA test.

Complication Type	+	−	
Total Implant Loss	20 implants (6.5%)	524 implants (96.3%)	544 implants
Early Implant Loss	6 implants (1.1%)	538 implants (98.9%)	544 implants
Delayed Implant Loss	14 implants (2.6%) *	524 implants (96.3%)	538 implants
Peri-implantitis	78 implants (14.5%) 34 patients (30.6%)	460 implants (85.5%) * 77 patients (69.3%)	538 implants 111 patients
History of previous periodontitis	46 patients (41.4%)	65 patients (58.5%)	111 patients
History of previous periodontitis in patients with peri-implantitis	20 patients (58.8%)	14 patients (41.7%)	*p* = 0.01350 ^#^
Smoking/Non-Smoking	1.92 ± 0.55	1.72 ± 0.50	*p* = 0.0017 ^&^
Peri-implantitis smoking	24 patients (70.5%)	10 patients (29.4%)	*p* = 0.00481 ^#^
Mean marginal bone loss	1.82 mm (SD = 0.54 mm.)		
Previous history of periodontitis	1.92 ± 0.50	1.75 ± 0.55	*p* = 0.0088 ^&^
Technical complications	76 implants (14.1%)	462 implants (85.8%)	538 implants

## Data Availability

If you want any additional information, you can consult the authors.

## Abbreviations

BIC	Bone Implant Contact
ASA	Sociedad Americana de Anestesiología
Ncm	Newtons-centimeter
CP	Commercially Pure titanium
